# Socioeconomic status and long-term health behaviour maintenance after non-communicable disease diagnosis: a multicohort study

**DOI:** 10.1186/s12916-025-04493-1

**Published:** 2025-11-26

**Authors:** Sunyi Wang, Yue Zhang, Yaguan Zhou, Mika Kivimäki, Xiaolin Xu

**Affiliations:** 1 School of Public Health, The Second Affiliated Hospital, Zhejiang University School of Medicine, Hangzhou, China; 2 The Key Laboratory of Intelligent Preventive Medicine of Zhejiang Province, Hangzhou, Zhejiang China; 3 UCL Brain Sciences, University College London, London, UK; 4 Clinicum, University of Helsinki, Helsinki, Finland; 5 School of Public Health, Faculty of Medicine, The University of Queensland, Brisbane, Australia

**Keywords:** Noncommunicable diseases, Socioeconomic inequalities, Healthy lifestyle, Physical inactivity, Smoking, Behaviours maintenance, Multicohort study

## Abstract

**Background:**

People with lower socioeconomic status (SES) have a higher incidence of non-communicable diseases (NCDs), but socioeconomic differences in the long-term maintenance of health behaviours after NCD diagnosis remain unclear. This study examined whether favourable health behaviours were maintained differently by SES over a 4-year follow-up.

**Methods:**

This prospective multicohort study pooled individual-level longitudinal data from five studies across 17 countries in Europe, the USA and East Asia between 2002 and 2021. Participants who were diagnosed with one or more major NCDs (diabetes, cardiovascular disease, chronic lung disease and cancer) and had favourable behaviours (being physically active and non-smokers, two widely recommended modifiable lifestyle factors) within the first 2 years after the diagnosis (baseline) were included, and were followed up for another 4 years to assess maintenance of health behaviours. SES was constructed using educational level and total household wealth (THW). Logistic regression models were used to analyse pooled multicohort data.

**Results:**

A total of 8518 participants were included, of whom 6629 were physically active, 7588 were non-smokers and 5699 were both physically active and non-smokers after the NCD diagnosis. During 4 years of follow-up, 1477 (22.3%) participants failed to maintain physical activity, and 154 (2.0%) became smokers. Educational level, THW and the summed SES score showed dose-response relationships with becoming physically inactive or smokers. For example, compared to participants with the high summed SES score, those with the low score were more likely to become physically inactive (odds ratio [OR]: 3.28, 95% confidence interval [CI]: 2.51–4.28) and smokers (OR: 2.91, 95% CI: 1.36–6.20). This was also seen in those who were both physically active and non-smokers at baseline, with low summed SES score having higher odds of becoming physically inactive but maintaining non-smokers (OR: 3.62, 95% CI: 2.71–4.84).

**Conclusions:**

These findings highlight a pronounced socioeconomic gradient in long-term maintenance of health behaviours after NCD diagnosis, with the low summed SES score more than doubling the risk of becoming physically inactive or smokers within 4 years. Socioeconomic position should be considered in strategies for managing individuals with non-communicable diseases.

**Supplementary Information:**

The online version contains supplementary material available at 10.1186/s12916-025-04493-1.

## Background

Population ageing is driving a continuous increase in the prevalence of non-communicable diseases (NCDs) [1]. According to 2022 data from the World Health Organization (WHO), the four major NCDs—diabetes, cardiovascular disease, chronic lung disease and cancer—account for approximately 41 million deaths annually, representing three-fourths of worldwide mortality [2]. The substantial impact of NCDs on public health has become a significant global concern, and the socioeconomic gradient—the lower the socioeconomic position, the worse the health—is widely recognized [3, 4].

It is well-established that lifestyle behaviours play important roles in the progression and prognosis of several major NCDs [5–7]. The teachable moment model suggests that health crises (e.g. NCD diagnosis) can create transient opportunities for behaviour change by increasing perceived susceptibility and emotional arousal [8], while long-term maintenance is a highly complex process and is influenced by a multitude of individual and environmental determinants, and thus requires strategies that extend beyond short-term motivational triggers. In this context, lifestyle-based health interventions are important not only for primary prevention, but also for secondary prevention and sustained management of NCDs [9]. The WHO guidelines recommend maintaining health behaviours to prevent the development of NCDs, including reducing tobacco use and maintaining physical activity [10]. Despite this growing public health recognition, modifying lifestyle behaviours in people diagnosed with NCDs remains challenging [11–15]. Previous studies have observed that only 15.4 to 48.0% of people quit smoking or increased physical activity volume after the diagnosis of NCDs. However, these studies had relatively shorter follow-up periods, with the median follow-up time ranging from 6 months to 3 years after the diagnosis of NCDs. In the long run, people can benefit from the long-term maintenance of health behaviours. Once they do not maintain health behaviours, these health benefits could be weakened or lost, according to a systematic review [16]. To date, however, few studies have investigated whether favourable behavioural changes continue over several years after NCD diagnosis.

Socioeconomic status (SES) is an important social determinant associated with health outcomes [17, 18]. Studies have shown that low SES is associated with higher risks of cardiovascular diseases, metabolic diseases, their complications and even premature mortality [17, 19–22]. Additionally, a previous study has already verified that lower levels of education and income are associated with a significantly higher prevalence of health risk behaviours, including physical inactivity and smoking [23]. However, most prior studies used cross-sectional designs or single-country data, which preclude assessing the impact of SES on temporal dynamics of health behavioural changes and limit generalizability across diverse socioeconomic and policy contexts. A recent multi-cohort study found that lower SES was associated with a reduced likelihood of health behavioural changes before and after the diagnosis of NCDs [24]. Nevertheless, this multi-cohort evidence did not capture the long-term maintenance of these favourable changes, leaving the role of SES in sustaining health behaviours after NCD diagnosis largely unexplored.

Using harmonized individual-level data from five prospective cohorts spanning 17 countries across Europe, the USA and East Asia, the present study addressed the following specific research questions: (1) To what extent individuals maintain health behaviours (physical activity and non-smoking) following NCD diagnosis; (2) How SES indicators (educational level, total household wealth and a composite SES) are associated with the maintenance of these behaviours.

## Methods

### Study design and participants

This multicohort study pooled individual-level data from five prospective cohort studies between 2002 and 2021 across 17 countries based on the Global Aging, Health and Policy program [25]: the US Health and Retirement Study (HRS), the Survey of Health, Ageing and Retirement in Europe (SHARE), the English Longitudinal Study on Ageing (ELSA), the China Health and Retirement Longitudinal Study (CHARLS) and the Korean Longitudinal Study of Aging (KLoSA). All these five studies were designed to ensure comparability and followed harmonized protocols in several key domains: nationally representative sampling of community-dwelling adults aged 45 years and older; standardized questionnaires on demographic characteristics, socioeconomic status, lifestyle behaviours and health status; and data collection every two years using comparable instruments and procedures. In the current study, we utilized waves 7–15 of HRS (conducted from 2004 to 2021), waves 1–9 of ELSA (conducted from 2002 to 2019), waves 1–8 of SHARE (conducted from 2004 to 2020), waves 1–5 of CHARLS (conducted from 2011 to 2021) and waves 1–8 of KLoSA (conducted from 2006 to 2021).

**Fig. 1:**
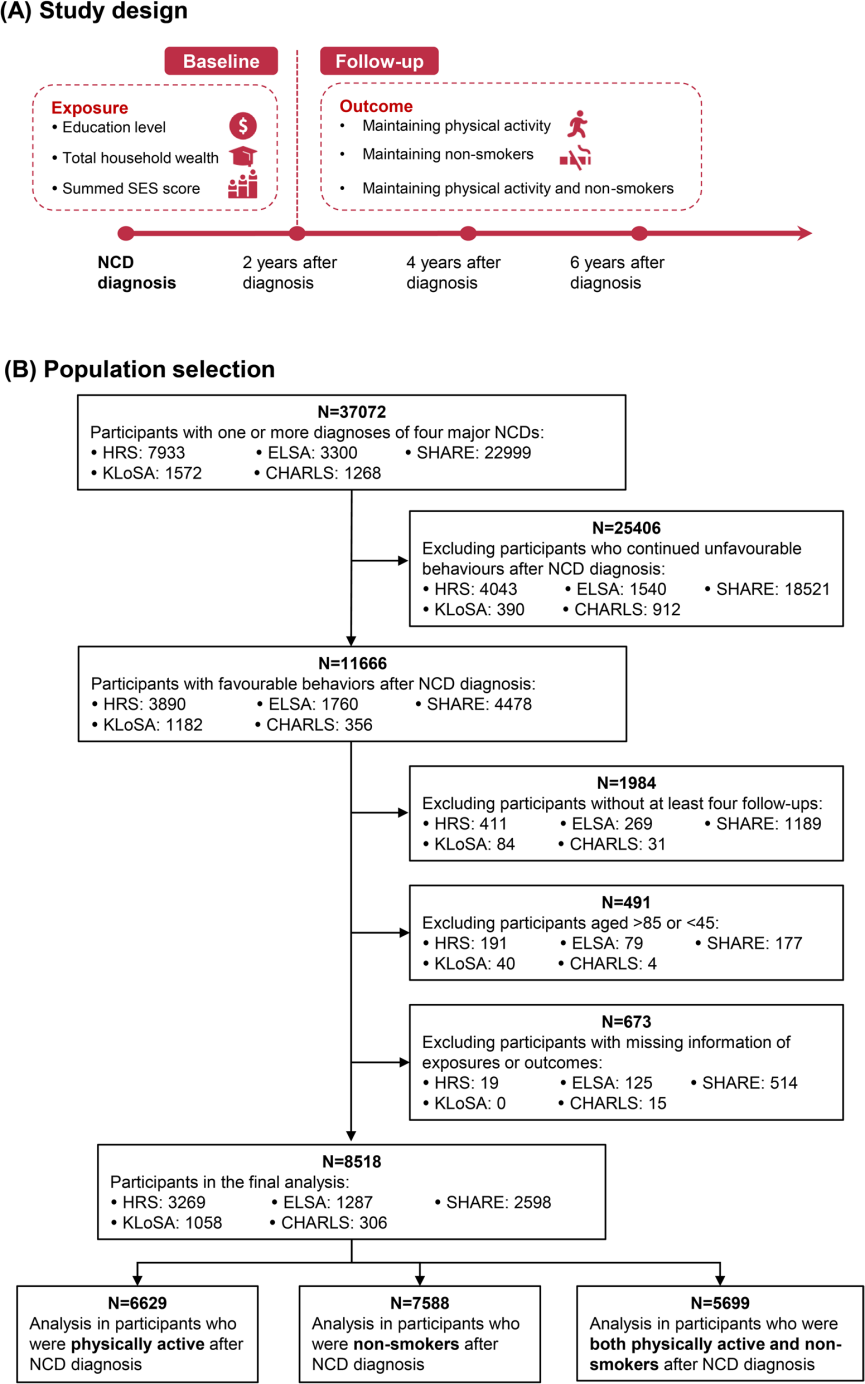
Study design and flow chart of the population selection process. **A** The study design. **B** The population selection. *SES* socioeconomic status; *HRS* the US Health and Retirement Study; *ELSA* English Longitudinal Study on Ageing; *SHARE*, Survey of Health, Ageing and Retirement in Europe; *CHARLS* China Health and Retirement Longitudinal Study; *KLoSA* Korean Longitudinal Study of Aging

Figure 1 illustrates the study design and population selection process. The analysis focused on middle-aged and older participants (≥ 45 years) who had health behaviours (being physically active or non-smokers) after the diagnosis of one or more self-reported major NCDs (diabetes, cardiovascular diseases, chronic lung diseases and cancers, *n* = 11666), which account for 50% of disability-adjusted life years [26] and are targeted in the WHO Global Action Plan [27]. Each participant was observed over five waves: the baseline was set at the first 2 years post-diagnosis of the NCDs, and participants were then followed up for the subsequent two surveys. After excluding participants without information for at least four follow-up survey, aged > 85 years, or those with missing data on exposures or outcomes, 8518 participants remained for the final analysis. Among these, 6629 participants were physically active, 7588 were non-smokers and 5699 were both physically active and non-smokers at baseline.

Our follow-up distinguished between participants who maintained health behaviours over the subsequent 4 years and those who became physically inactive (among those initially active) or became current smokers (among those initially non‑smokers) during this period. Among participants who were both physically active and non‑smoking at baseline, we defined four mutually exclusive outcome categories at follow‑up: maintaining physically active and non‑smokers; becoming physically inactive solely; becoming smokers solely; becoming both physically inactive and smokers.

### Assessment of socioeconomic status

The main exposure of this study is SES, measured at baseline using educational level and total household wealth (THW), consistent with our previous studies [24, 28, 29]. Educational level at baseline was categorized as primary (scored 0), secondary (scored 1) and tertiary (scored 2), while THW was divided into quartiles representing the lowest (quartile 1, scored 0) to the highest (quartile 4, scored 3). Due to different income levels in various countries’ currency units (13 European countries in the SHARE study have been harmonized into euros), the four categories of THW were calculated separately in each cohort at the baseline and then harmonized (Details in Additional file 1: Table S1).

Although previous evidence showed that educational level and THW are generally correlated [30], the correlation is insufficient to use one as a proxy for the other (Additional file 1: Table S2). Therefore, the composite SES was constructed in two ways as in previous studies [24, 28, 29]: (1) combining 3 categories of educational and 4 categories of THW into 12 groups (3*4); (2) summing scores for educational level and THW quartiles to obtain a summed SES score (0–5), which was further grouped into four categories: low SES (0), lower-middle (1–2), upper-middle (3–4) and high SES (5).

### Assessment of lifestyle behaviours

The outcomes of interest were the maintenance of health behaviours during follow-up. Based on the harmonized data, participants reporting engagement in physical activity more than once per week were considered physically active, and those reporting no smoking at follow-up were considered non-smokers (Details in Additional file 1: Table S1).

### Covariates

Baseline characteristics collected included age, sex (male/female), country, study (HRS, ELSA, SHARE, CHARLS, KLoSA), marital status (married/partnered, separated/divorced, widowed and never married), alcohol drinking status (less than weekly drinking, weekly drinking or more), body mass index (BMI; underweight, < 18.5; healthy weight, 18.5–24.9; overweight, 25–29.9 and obesity, ≥ 30.0 [31]), and the disease history of hypertension, psychological disorder and major NCDs. Hypertension, psychological disorders and major NCDs were categorized as ever and never diagnosed according to participants’ self-reported disease history. According to the previous evidence [24, 32, 33], age, sex and study were confounders and included in the final analysis model. All variables included in the final analysis model had complete data with no missing values.

### Statistical analysis

Baseline characteristics of participants were summarized by SES and behavioural maintenance. Continuous variables were described as mean (standard deviation, SD), and categorical variables were as counts (percentages), with differences between groups evaluated using *t* test and Pearson’s χ^2^ test, respectively.

We used logistic regression models to assess the association of independent SES indicators (educational level, THW), 4 categorized summed SES scores (low, lower-middle, upper-middle, high) and 12 SES combinations (3 educational levels × 4 THW quartiles) with the maintenance of favourable behaviours by estimating odds ratios (ORs) and 95% confidence intervals (CIs). To examine indicator-specific associations, educational level and THW were analysed in separate models, with mutual adjustment (i.e. the model for education included an adjustment for THW, and the model for THW included an adjustment for education). Models for the summed SES score did not simultaneously adjust for educational level or THW to avoid multicollinearity. We adjusted for the smoking status in the analysis of physical activity, and vice versa. For participants who were both physically active and non‑smoking, we used multinomial logistic regression to model the four‑category outcomes, with results reported as multinomial odds ratios (mORs).

Besides, a series of additional analyses was conducted to test the robustness of the results. Meta-analyses were performed to pool the study-specific ORs of SES on the maintenance of favourable behaviours using random effects models and assessed the between-study heterogeneity with the *I*^2^ statistic. We further conducted subgroup analyses to assess the association of SES with the maintenance of favourable behaviours stratified by age, sex and pre‑diagnosis behaviour status. Third, sensitivity analyses were performed by replacing the outcomes with individual NCDs, and by evaluating behaviours at both the shorter-term (2-year) and longer-term (4-year) follow-up intervals.

Statistical analyses were performed using SAS (version 9.4, SAS Institute Inc., NC, USA) and R software (version 4.3.3, Vienna, Austria). Significance tests were evaluated at the 0.05 level using two-sided tests.

## Results

### Baseline characteristics of participants

A total of 8518 participants were included in the present study. Among these participants, 3885 (45.6%) were from European countries, 3269 (38.4%) were from the USA and 1364 (16.0%) were from East Asian countries (China, South Korea). The mean (SD) age of participants was 68.7 (8.4) years, and 4841 (56.8%) participants were females (Additional file 1: Table S3). Compared to participants with high SES, those with low SES were more likely to be older, women, with no partner, consume less alcohol and have a higher prevalence of hypertension, diabetes, cardiovascular diseases, chronic lung diseases and cancers (*P* < 0.05) (Table 1).

**Table 1:** Characteristics of participants after NCD diagnosis by socioeconomic status (*n* = 8518)

**Characteristics**^**a**^	**Overall** **(*n*** = **8518)**	**Summed SES score**^**b**^				***P* value**^**c**^
		**Low** **(*n*** = **959)**	**Lower-middle** **(*n*** = **3468)**	**Upper-middle** **(*n*** = **3078)**	**High** **(*n*** = **1013)**	
**Age, mean (SD)**	68.7 (8.4)	69.7 (9.1)	68.5 (8.5)	68.5 (8.2)	68.5 (8.0)	< 0.001
**Age (years)**						< 0.001
45–54	338 (4.0)	41 (4.3)	159 (4.6)	112 (3.6)	26 (2.6)	
55–64	2551 (29.9)	268 (27.9)	1043 (30.1)	933 (30.3)	307 (30.3)	
65–74	3296 (38.7)	316 (33.0)	1314 (37.9)	1219 (39.6)	447 (44.1)	
75–85	2333 (27.4)	334 (34.8)	952 (27.5)	814 (26.4)	233 (23.0)	
**Sex**						< 0.001
Male	3677 (43.2)	317 (33.1)	1320 (38.1)	1476 (48.0)	564 (55.7)	
Female	4841 (56.8)	642 (66.9)	2148 (61.9)	1602 (52.0)	449 (44.3)	
**Marital status**						< 0.001
Married/partnered	5793 (68.0)	489 (51.0)	2169 (62.5)	2319 (75.3)	816 (80.6)	
Separated/divorced	827 (9.7)	132 (13.8)	386 (11.1)	244 (7.9)	65 (6.4)	
Widowed	1531 (18.0)	285 (29.7)	746 (21.5)	400 (13.0)	100 (9.9)	
Never married	341 (4.0)	52 (5.4)	153 (4.4)	109 (3.5)	27 (2.7)	
Missing	26 (0.3)	1 (0.1)	14 (0.4)	6 (0.2)	5 (0.5)	
**Body mass index**						< 0.001
Underweight	148 (1.7)	30 (3.1)	71 (2.0)	39 (1.3)	8 (0.8)	
Normal weight	2468 (29.0)	250 (26.1)	958 (27.6)	912 (29.6)	348 (34.4)	
Overweight	2797 (32.8)	280 (29.2)	1,080 (31.1)	1067 (34.7)	370 (36.5)	
Obesity	2089 (24.5)	234 (24.4)	916 (26.4)	728 (23.7)	211 (20.8)	
Missing	1016 (11.9)	165 (17.2)	443 (12.8)	332 (10.8)	76 (7.5)	
**Drinking status**						< 0.001
Less than weekly drinking	4805 (56.4)	651 (67.9)	2228 (64.2)	1557 (50.6)	369 (36.4)	
Weekly drinking or more	3519 (41.3)	254 (26.5)	1162 (33.5)	1466 (47.6)	637 (62.9)	
Missing	194 (2.3)	54 (5.6)	78 (2.2)	55 (1.8)	7 (0.7)	
**Hypertension**	5367 (63.0)	644 (67.2)	2295 (66.2)	1879 (61.0)	549 (54.2)	< 0.001
**Psychological disorders**	1315 (15.4)	177 (18.5)	544 (15.7)	465 (15.1)	129 (12.7)	0.051
**Major NCDs**						
Diabetes	2822 (33.1)	352 (36.7)	1260 (36.3)	945 (30.7)	265 (26.2)	< 0.001
Cardiovascular diseases	3712 (43.6)	472 (49.2)	1,513 (43.6)	1324 (43.0)	403 (39.8)	0.002
Chronic lung diseases	1163 (13.7)	158 (16.5)	524 (15.1)	380 (12.3)	101 (10.0)	< 0.001
Cancer	1778 (20.9)	136 (14.2)	593 (17.1)	743 (24.1)	306 (30.2)	< 0.001

^a^Characteristics was assessed at the first 2-year post-diagnosis of the NCDs. *NCDs* non-communicable diseases, *SES* socioeconomic status. Data are *n* (%) unless otherwise indicated

^b^Socioeconomic status was constructed as the summed score (from 0 to 5) of education level (0, 1, or 2) and THW quartiles (0, 1, 2, or 3), and categorized into four groups of low (0), lower-middle (1–2), upper-middle (3–4), and high (5)

^c^*T* test and Chi-squared test were used to compare differences across groups

### Long-term maintenance of health behaviours after NCD diagnosis

**Fig. 2:**
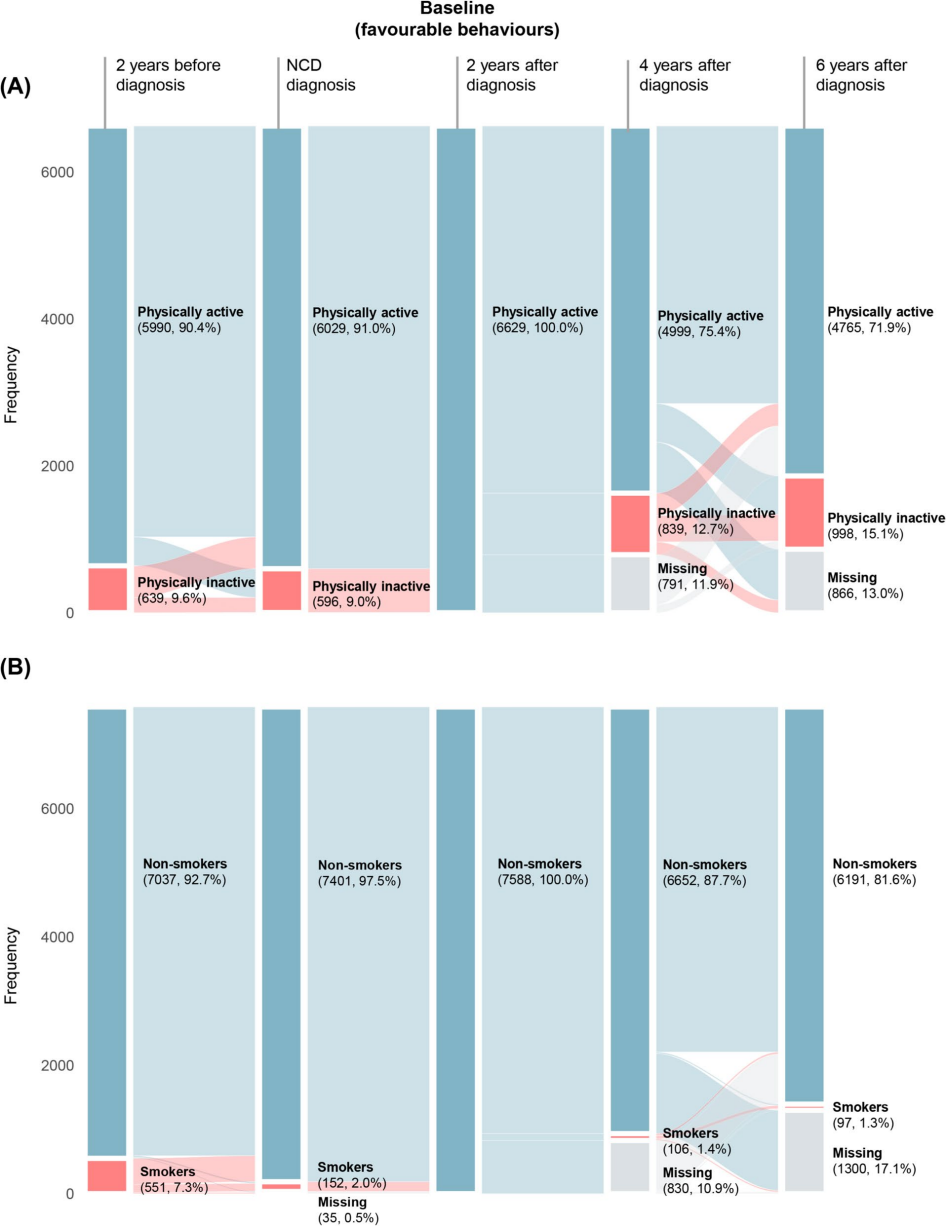
Sankey diagram of the change of **A** physical activity and **B** smoking across the five waves. *NCD* non-communicable disease

Of all 6629 participants who were physically active after NCD diagnosis, 1477 (22.3%) became physically inactive during follow-up. Among these, 1145 had been physically active both before and after the NCD diagnosis but became physically inactive during follow-up, while 332 were physically inactive before diagnosis and physically active after NCD diagnosis but eventually failed to maintain during follow-up. Of the 7588 non-smokers at baseline, 154 (2.0%) became smokers during follow-up. Among these, 65 initiated smoking, while 89 were relapsing smokers (Additional file 1: Table S4). The distribution of specific behaviour changes over five waves is shown in Fig. 2.

### Association between SES indicators and the long-term maintenance of physical activity

Participants with lower educational levels or in lower quartiles of THW had higher odds of becoming physically inactive during follow-up, compared to those with higher educational levels or in higher THW quartiles (Table 2). For example, among all participants who were physically active after NCD diagnosis, the odds of becoming physically inactive were 1.94-fold (primary education vs*.* tertiary education, 95% CI 1.64–2.30) and 1.74-fold (lowest THW quartile vs*.* highest THW quartile, 95% CI 1.46–2.08) higher among participants with low educational level or low THW, respectively. The summed SES score showed a dose–response relationship with the risk of becoming physically inactive over the follow-up (*p* for trend < 0.001), with progressively higher odds from high to low SES. Individuals with a low summed SES score had 3.28 times higher odds (95% CI 2.51–4.28) of becoming physically inactive. We also found that the odds of not maintaining physically active were highest in the combination of primary educational level and the lowest quartile of THW (Additional file 1: Fig. S1).

**Table 2:** Associations between socioeconomic indicators and becoming physically inactive during follow-up (*N* = 6629)

	**Maintaining physically active (*n*** = **5152)**		**Becoming physically inactive (*n*** = **1477)**	
	Events (%)	OR (95% CI)	Events (%)	OR (95% CI)
**Educational level**				
Primary	1451 (70.61)	1.00 (Ref)	604 (29.39)	1.94 (1.64, 2.30)
Secondary	1933 (77.07)	1.00 (Ref)	575 (22.93)	1.57 (1.34, 1.85)
Tertiary	1768 (85.58)	1.00 (Ref)	298 (14.42)	1.00 (Ref)
*P* for trend				< 0.001
**THW**				
Quartile 1 (Lowest)	974 (69.87)	1.00 (Ref)	420 (30.13)	1.74 (1.46, 2.08)
Quartile 2	1222 (75.34)	1.00 (Ref)	400 (24.66)	1.35 (1.14, 1.61)
Quartile 3	1359 (80.27)	1.00 (Ref)	334 (19.73)	1.07 (0.90, 1.27)
Quartile 4 (Highest)	1597 (83.18)	1.00 (Ref)	323 (16.82)	1.00 (Ref)
*P* for trend				< 0.001
**Summed SES score**				
Low (0)	411 (66.40)	1.00 (Ref)	208 (33.60)	3.28 (2.51, 4.28)
Lower-middle (1–2)	1836 (72.77)	1.00 (Ref)	687 (27.23)	2.55 (2.04, 3.19)
Upper-middle (3–4)	2095 (81.61)	1.00 (Ref)	472 (18.39)	1.57 (1.25, 1.96)
High (5)	810 (88.04)	1.00 (Ref)	110 (11.96)	1.00 (Ref)
*P* for trend				< 0.001

*THW* total household wealth, *SES* socioeconomic status, *Ref* reference. Summed SES-score was constructed as the summed score (ranging from 0 to 5) of educational level (0, 1, or 2) and THW quartiles (0, 1, 2, or 3), and categorized into four groups of low (0), lower-middle (1–2), upper-middle (3–4), and high SES (5). Reference groups: tertiary educational level, highest total household wealth, and high summed SES score. Models were adjusted for age, sex, study, and smoking status at baseline

### Association between SES indicators and the long-term maintenance of non-smokers

Among participants who were non-smokers after NCD diagnosis, the odds of becoming smokers during follow-up were higher in those with lower educational levels and lower THW quartiles. Specifically, the odds were 1.35 times higher for participants with primary education vs. tertiary education (95% CI 0.84–2.17), and 1.93 times higher for those in the lowest vs. the highest THW quartile (95% CI 1.18–3.15). Furthermore, a dose–response relationship was also observed between the summed SES score and becoming current smokers over the follow-up, ORs of 2.91 (95% CI 1.36–6.20) for low, 2.29 (95% CI 1.19–4.40) for lower-middle and 1.83 (95% CI 0.95–3.52) for upper-middle compared to high SES (Table 3). Similar results were found in the combinations of educational levels and the quartiles of THW (Additional file 1: Fig. S1).

**Table 3:** Associations between socioeconomic indicators and becoming smokers during follow-up (*N* = 7588)

	**Maintaining non-smokers (*n*** = **7434)**		**Becoming smokers (*n*** = **154)**	
	Events (%)	OR (95% CI)	Events (%)	OR (95% CI)
**Educational level**				
Primary	2768 (97.91)	1.00 (Ref)	59 (2.09)	1.35 (0.84, 2.17)
Secondary	2599 (97.74)	1.00 (Ref)	60 (2.26)	1.34 (0.86, 2.08)
Tertiary	2067 (98.33)	1.00 (Ref)	35 (1.67)	1.00 (Ref)
*P* for trend				0.248
**THW**				
Quartile 1 (Lowest)	1564 (97.26)	1.00 (Ref)	44 (2.74)	1.93 (1.18, 3.15)
Quartile 2	1932 (97.67)	1.00 (Ref)	46 (2.33)	1.64 (1.02, 2.65)
Quartile 3	1896 (98.34)	1.00 (Ref)	32 (1.66)	1.14 (0.69, 1.88)
Quartile 4 (Highest)	2042 (98.46)	1.00 (Ref)	32 (1.54)	1.00 (Ref)
*P* for trend				0.003
**Summed SES score**				
Low (0)	801 (97.45)	1.00 (Ref)	21 (2.55)	2.91 (1.36, 6.20)
Lower-middle (1–2)	2992 (97.81)	1.00 (Ref)	67 (2.19)	2.29 (1.19, 4.40)
Upper-middle (3–4)	2717 (98.02)	1.00 (Ref)	55 (1.98)	1.83 (0.95, 3.52)
High (5)	924 (98.82)	1.00 (Ref)	11 (1.18)	1.00 (Ref)
*P* for trend				0.003

*THW* total household wealth, *SES* socioeconomic status, *Ref* reference. Summed SES-score was constructed as the summed score (ranging from 0 to 5) of educational level (0, 1, or 2) and THW quartiles (0, 1, 2, or 3), and categorized into four groups of low (0), lower-middle (1–2), upper-middle (3–4), and high SES (5). Reference groups: tertiary educational level, highest total household wealth, and high summed SES score. Models were adjusted for age, sex, study, and physical activity status at baseline

### Association between SES indicators and the long-term maintenance of both physical activity and non-smokers

Considering both physically active and non-smokers at baseline, the associations between SES indicators and unfavourable behaviour maintenance were still significant in most groups. Educational level showed a significant dose‑response relationship with becoming physically inactive only (*p* for trend < 0.001), with participants having primary education showing 2.04 times higher odds (95% CI 1.70–2.46) compared to those with tertiary education. A similar pattern was observed for dual adverse outcomes (becoming both physically inactive and current smokers). THW demonstrated consistent associations, with the lowest quartile showing 1.71 times higher odds (95% CI 1.41–2.07) of becoming physically inactive only compared to the highest quartile. Compared to individuals with a high summed SES score, those with a low score had mOR of 3.62 (95% CI 2.71–4.84) for becoming physically inactive only (Table 4).

**Table 4:** Associations between socioeconomic indicators and maintenance of favourable behaviours during follow-up (*N* = 5699)

	**Maintaining physically active and non-smokers (*n*** = **4355)**		**Becoming physically inactive solely (*n*** = **1230)**		**Becoming smokers solely (*n*** = **96)**		**Becoming both physically inactive and smokers (*n*** = **18)**	
	Events (%)	mOR (95% CI)	Events (%)	mOR (95% CI)	Events (%)	mOR (95% CI)	Events (%)	mOR (95% CI)
**Educational level**								
Primary	1190 (68.51)	1.00 (Ref)	512 (29.48)	2.04 (1.70, 2.46)	27 (1.55)	1.20 (0.68, 2.12)	8 (0.46)	9.80 (1.15, 83.45)
Secondary	1624 (75.99)	1.00 (Ref)	466 (21.81)	1.56 (1.30, 1.86)	38 (1.78)	1.16 (0.69, 1.92)	9 (0.42)	8.35 (1.02, 68.03)
Tertiary	1541 (84.44)	1.00 (Ref)	252 (13.81)	1.00 (Ref)	31 (1.70)	1.00 (Ref)	1 (0.05)	1.00 (Ref)
*P* for trend				< 0.001		0.525		0.031
**THW**								
Quartile 1 (Lowest)	739 (68.17)	1.00 (Ref)	320 (29.52)	1.71 (1.41, 2.07)	19 (1.75)	1.43 (0.76, 2.69)	6 (0.55)	2.47 (0.59, 10.28)
Quartile 2	1015 (73.34)	1.00 (Ref)	333 (24.06)	1.33 (1.11, 1.60)	31 (2.24)	1.77 (1.02, 3.07)	5 (0.36)	1.63 (0.38, 6.99)
Quartile 3	1178 (78.80)	1.00 (Ref)	292 (19.53)	1.07 (0.89, 1.29)	21 (1.40)	1.02 (0.56, 1.85)	4 (0.27)	1.23 (0.27, 5.55)
Quartile 4 (Highest)	1423 (81.97)	1.00 (Ref)	285 (16.42)	1.00 (Ref)	25 (1.44)	1.00 (Ref)	3 (0.17)	1.00 (Ref)
*P* for trend				< 0.001		0.081		0.180
**Summed SES score**								
Low (0)	304 (63.07)	1.00 (Ref)	169 (35.06)	3.62 (2.71, 4.84)	7 (1.45)	1.82 (0.69, 4.79)	2 (0.41)	/
Lower-middle (1–2)	1514 (71.62)	1.00 (Ref)	553 (26.16)	2.52 (1.98, 3.20)	37 (1.75)	1.83 (0.92, 3.65)	10 (0.47)	/
Upper-middle (3–4)	1802 (79.70)	1.00 (Ref)	412 (18.22)	1.63 (1.28, 2.07)	41 (1.81)	1.62 (0.82, 3.18)	6 (0.27)	/
High (5)	735 (87.29)	1.00 (Ref)	96 (11.40)	1.00 (Ref)	11 (1.31)	1.00 (Ref)	0 (0.00)	/
*P* for trend				< 0.001		0.119		/

*THW* Total household wealth, *SES* Socioeconomic status, *mOR* multinomial odds ratio, *Ref* reference. Summed SES-score was constructed as the summed score (ranging from 0 to 5) of educational level (0, 1, or 2) and THW quartiles (0, 1, 2, or 3), and categorized into four groups of low (0), lower-middle (1–2), upper-middle (3–4), and high SES (5). Reference groups: tertiary educational level, highest total household wealth, and high summed SES score. Models were adjusted for age at baseline, sex, and study

### Supplementary and sensitivity analyses

**Fig. 3:**
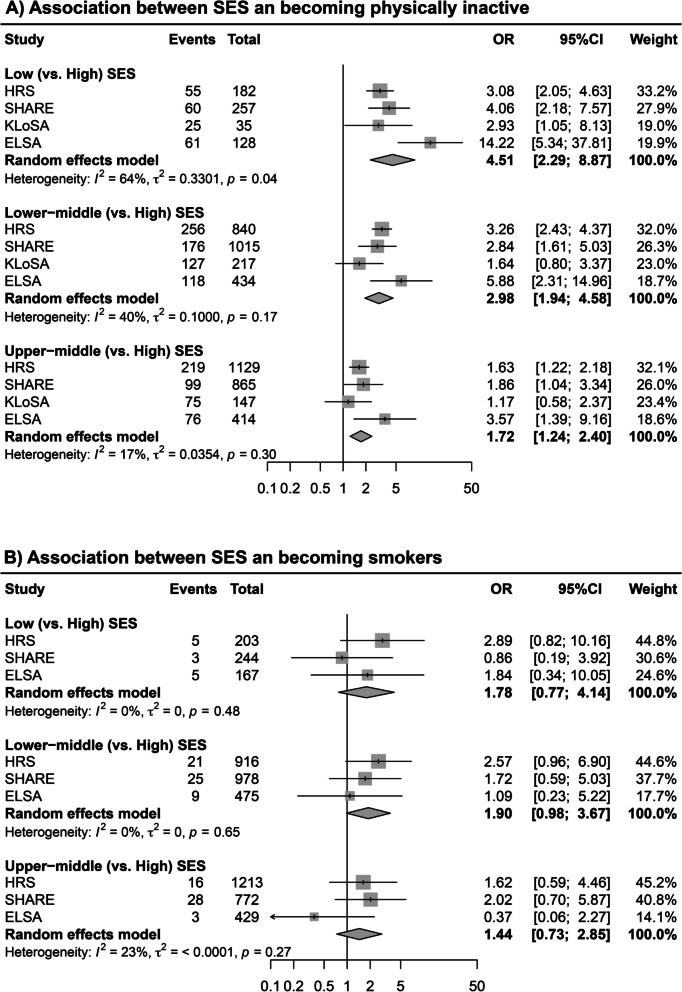
Forest plot of study-specific ORs of the associations between SES and maintenance of favourable behaviours. The results were adjusted for age and sex. *SES* socioeconomic status, *HRS* the US Health and Retirement Study, *SHARE* Survey of Health, Ageing and Retirement in Europe; *KLoSA*, Korean Longitudinal Study of Aging, *ELSA*, English Longitudinal Study on Ageing

The pooled ORs for the association between summed SES score and becoming physically inactive in the meta-analysis (Fig. 3) were 4.51 (low vs. high; 95% CI 2.29–8.87), 2.98 (low-middle vs. high; 95% CI 1.94–4.58) and 1.72 (upper-middle vs. high; 95% CI 1.24–2.40). For smoking behaviour, the pooled ORs for the association between summed SES score and initiating or relapsing into smoking presented similar patterns (Additional file 1: Table S5, Fig. S2).

Subgroup analyses stratified by age, sex and pre‑diagnosis behaviours showed similar trends in the association between SES and the maintenance of health behaviours. (Additional file 1: Table S6, S7). These associations were consistently observed in sensitivity analyses (Additional file 1: Table S8, S9).

## Discussion

This multi-cohort study, which pooled individual-level data from 8518 participants aged 45–85 years across 17 countries is apparently the first large-scale multinational study to demonstrate a pronounced socioeconomic gradient in the long-term maintenance of health behaviours after NCD diagnosis. Among participants who were physically active following the NCD diagnosis, nearly one-fifth were unable to maintain this health behaviour over the subsequent four years. Approximately one in six participants who quit smoking after disease onset eventually relapsed. Low socioeconomic status, measured by low educational level or low total household wealth, more than doubled the risk of becoming physically inactive or smoking.

Our findings complement previous research on socioeconomic differences in health. For middle-aged and elderly participants, NCD diagnosis may serve as a motivation to lifestyle modification; however, sustaining health behaviours over time is often more challenging [34–36]. A multi-cohort study found that only 40% of participants were able to increase physical activity, and 31% of smokers were able to quit within two years after NCD diagnosis [24]. In a randomized controlled trial, nearly 30% of participants who were diagnosed with cancers relapsed into smoking within 2 months of quitting, and 40% relapsed after 12 months of quitting [37]. Our study extended previous studies by employing substantially longer observation periods extending up to 6 years after the NCD diagnosis for examining the health behaviours. These findings highlight that the ability to sustain health behaviours is closely linked to individuals’ socioeconomic resources. This must be more effectively considered when developing intervention strategies to support the long-term maintenance of healthy lifestyle behaviours among patients with NCDs in clinical practice.

Several factors may contribute to the difficulty of maintaining a healthy lifestyle. With the progression of chronic diseases and increasing age, people often experience a decline in physical capacity, limiting their ability to engage in physical activities [38]. Nicotine addiction, increasing the likelihood of replacement therapy and decreasing the likelihood of quitting, is influenced by a complex interaction of factors, such as psychological control, tobacco access and sociocultural influences from family and peers [39]. Smokers diagnosed with diabetes have been found to have lower cessation rates and greater difficulty managing blood glucose and body weight after quitting [40].

Building on these observations, previous evidence has shown that many determinants cluster by socioeconomic position [17, 41, 42], with health disadvantages being more evident among lower SES groups post-diagnosis. Our study extended previous studies by focusing on the long-term maintenance of these behaviours, suggesting a dose–response relationship between educational level, household wealth and the odds of discontinuing health behaviours during follow-up [43–45]. The underlying mechanisms remain unclear. Generally, participants with higher educational levels have better health literacy, higher self-efficacy and better resources to maintain health behaviours because they are more capable of accessing, understanding and applying health-related knowledge [46]. Wealthier individuals have better access to social and health resources, clinical care and technologies, like smartphones and wearables, all of which can also support the long-term maintenance of health behaviours [47, 48]. By contrast, previous evidence found that older adults with low SES may have limited health information and lower awareness of the benefits of physical activity [49]. Gaps in disease knowledge and health literacy, fewer social supports, time and financial constraints and less supportive neighbourhood environments may further impede long‑term behaviour change [50, 51].

The associations between SES and failure to maintain physical activity were particularly strong among females aged 55–75 years. Compared with participants with other NCDs, participants with cancer were more likely to become physically inactive due to exercise limitations in the functions of the lungs. SES was significantly associated with higher odds of relapsing smoking in males aged 55–65 years and in participants diagnosed with cardiovascular diseases, chronic lung disease or diabetes than in those with cancer, which usually has a shorter life expectancy. Previous studies on sex differences in smoking cessation are inconsistent and do not fully address these subgroups. For example, a global systematic review found no sex differences in persistent or relapsing smoking among smokers, but did not provide a comparison between socioeconomic groups [52]. Data from the Global Burden of Disease [53] suggested the need for sustained cessation efforts for both sexes, although this was not examined in relation to prevalent NCDs.

The strengths of our study include the combination of individual-level data from five large prospective population-based cohorts spanning 17 countries across Europe, the US and East Asia, enhancing robustness and supporting generalizability to middle‑ and high‑income countries; coordinated measurement enabling cross‑national comparability; and a longitudinal design capturing 4‑year maintenance of favourable behaviours after NCD diagnosis. There remain several limitations that should be noted. First, considering our analysis needed long-term follow-up and the oldest participants could be lost to follow-up, we excluded participants aged 85 years and older, which may limit our findings in such populations. Second, this study used self-reported data, which may introduce measurement errors and potentially cause non-differential bias and underestimate true associations. Third, the study did not account for chronic disease symptoms, subtypes, severity, treatment regimens or side effects, all of which could affect the maintenance of health behaviours. Fourth, due to the availability of relevant harmonised data, the measurement of SES only included educational level and household wealth, without considering other factors like occupation and ethnicity [54], and the behaviours assessed in this study were limited to physical activity and smoking. Fifth, the study used binary classifications for smoking and physical activity, neither capturing reductions in smoking frequency/amounts or e-cigarette use, nor evaluating increases in physical activity frequency or volume; this may lead to non‑differential misclassification and bias estimates toward the null, which need further exploration. Sixth, although we have adjusted for several known covariates in the statistical models, residual confounding remains a possibility in all observational research. In addition, while this study encompasses data from 17 countries, the sample predominantly represents middle- to high-income nations, which limits the generalizability to populations in lower-income or less-developed settings, where disparities in health outcomes and healthcare access are often more pronounced.

Our results provide important implications for the secondary prevention of NCDs. At the community level, targeted interventions such as developing accessible exercise facilities, implementing subsidized physical activity programs, and establishing community-based peer support groups for smoking cessation should be prioritized, particularly in disadvantaged neighborhoods. In clinical practice, care that is equity-oriented and patient-centred should be coupled with communication and decision aids tailored to health literacy levels, extended consultation time and proactive follow-up for lower-SES patients, to avoid unintentionally widening inequalities. From a policy perspective, governments and public health authorities need to implement SES-targeted strategies that directly reduce financial and time barriers and expand access to evidence‑based services (e.g. vouchers or conditional incentives, transport support, subsidized physical activity programmes and coverage of smoking‑cessation pharmacotherapies) with equitable resource allocation to disadvantaged communities. These multi-level interventions, addressing both individual behavioural factors and broader social determinants of health, are essential for improving quality of life, prolonging survival, and reducing health inequalities in populations with NCDs.

## Conclusions

Our study, which included populations from 17 countries, provides a longer-term observation of the health behaviours of participants diagnosed with NCDs compared to previous large-scale studies. Our findings indicate that the maintenance of health behaviours in individuals with NCDs is associated with socioeconomic circumstances across all these countries. At the social level, this evidence highlights the need for targeted interventions to address socioeconomic inequalities in secondary prevention of NCDs, ultimately improving health equity and quality of life.

## Supplementary Information

Additional file 1: Table S1. Harmonized strategies for key variables included in the present analyses. Table S2. Study-specific Spearman correlations between education and total household wealth. Table S3. Characteristics of participants in each study by country. Table S4. Characteristics of participants by health behaviour status before and after diagnosis of NCDs and maintenance. Table S5. Associations between the summed SES score and maintenance of behaviours in each study. Table S6. Subgroup analysis of associations between SES and maintenance of favourable behaviours by age and sex. Table S7. Subgroup analysis of associations between SES and maintenance of favourable behaviours by pre‑diagnosis behaviours. Table S8. Sensitivity analysis for the associations between SES and maintenance of favourable behaviours by individual NCDs. Table S9. Sensitivity analysis for the shorter- and longer-term associations between SES and maintenance of favourable behaviours. Fig. S1. Heatmaps of the associations of the 12 combinations of SES with maintenance of favourable behaviours. Fig. S2. Forest plot of study-specific ORs of the associations between SES and relapsing smoking in ex-smokers.

## Data Availability

The original data for this study are available on their respective websites: The Health and Retirement Study–HRS (https://hrs.isr.umich.edu/), the Survey of Health, Ageing and Retirement in Europe–SHARE (http://www.share-project.org/home0.html), the English Longitudinal Study of Ageing–ELSA (https://www.elsa-project.ac.uk), the China Health and Retirement Longitudinal Study–CHARLS (http://charls.pku.edu.cn/index/en.html), and the Korean Longitudinal Study of Aging–KLoSA (https://survey.keis.or.kr/eng/klosa/klosa01.jsp).

## Abbreviations

BMI: Body mass index
CHARLS: China Health and Retirement Longitudinal Study
CI: Confidence Interval
ELSA: English Longitudinal Study on Ageing
HRS: US Health and Retirement Study
KLoSA: Korean Longitudinal Study of Aging
MICE: Multiple imputation by chained equations
NCD: Non-communicable disease
OR: Odds ratio
mOR: Multinomial odds ratio
SD: Standard deviation
SES: Socioeconomic status
SHARE: Survey of Health, Ageing and Retirement in Europe
THW: Total household wealth
WHO: World Health Organization
