# Supplementary material for: Socioeconomic status and long-term health behaviour maintenance after non-communicable disease diagnosis: a multicohort study
Source: BMC Med. 2025 Nov 26;23:659. doi: 10.1186/s12916-025-04493-1 (PMC12659176; doi:10.1186/s12916-025-04493-1)
Supplement: Supplementary file 1 — Additional file 1: Table S1. Harmonized strategies for key variables included in the present analyses. Table S2. Study-specific Spearman correlations between education and total household wealth. Table S3. Characteristics of participants in each study by country. Table S4. Characteristics of participants by health behaviour status before and after diagnosis of NCDs and maintenance. Table S5. Associations between the summed SES score and maintenance of behaviours in each study. Table S6. Subgroup analysis of associations between SES and maintenance of favourable behaviours by age and sex. Table S7. Subgroup analysis of associations between SES and maintenance of favourable behaviours by pre‑diagnosis behaviours. Table S8. Sensitivity analysis for the associations between SES and maintenance of favourable behaviours by individual NCDs. Table S9. Sensitivity analysis for the shorter- and longer-term associations between SES and maintenance of favourable behaviours. Fig. S1. Heatmaps of the associations of the 12 combinations of SES with maintenance of favourable behaviours. Fig. S2. Forest plot of study-specific ORs of the associations between SES and relapsing smoking in ex-smokers. [file 12916_2025_4493_MOESM1_ESM.docx]

**Additional file 1**

**Table S1.** Harmonized strategies for key variables included in the present analyses.

**Table S2.** Study-specific Spearman correlations between education and total household wealth.

**Table S3.** Characteristics of participants in each study by country.

**Table S4.** Characteristics of participants by health behaviour status before and after diagnosis of NCDs and maintenance (N = 8,518).

**Table S5.** Associations between the summed SES score and maintenance of behaviours in each study.

**Table S6.** Subgroup analysis of associations between SES and maintenance of favourable behaviours by age and sex.

**Table S7.** Subgroup analysis of associations between SES and maintenance of favourable behaviours by pre‑diagnosis behaviours.

**Table S8**. Sensitivity analysis for the associations between SES and maintenance of favourable behaviours by individual NCDs.

**Table S9.** Sensitivity analysis for the shorter- and longer-term associations between SES and maintenance of favourable behaviours

**Fig. S1.** Heatmaps of the associations of the 12 combinations of SES with maintenance of favourable behaviours.

**Fig. S2**. Forest plot of study-specific ORs of the associations between SES and relapsing smoking in ex-smokers.

**Table S1.** Harmonized strategies for key variables included in the present analyses.

| **Variables** | **Harmonized values** | **Measurements in six studies** | | | | |
| --- | --- | --- | --- | --- | --- | --- |
|  |  | **HRS** | **ELSA** | **SHARE** | **KLoSA** | **CHARLS** |
| **Education level** | Primary | Less than upper secondary education | | | | |
|  | Secondary | Upper secondary, vocational training or some college | | | | |
|  | Tertiary | Tertiary education (college and above) | | | | |
| **Total household wealth** | Quartile 1 (low) | Sum of all wealth components (including residence, vehicles, saving accounts, etc.) minus other debts at the couple level (the respondent and spouse, if any) in local currencies | | | | |
|  | Quartile 2 |  |  |  |  |  |
|  | Quartile 3 |  |  |  |  |  |
|  | Quartile 4 (high) |  |  |  |  |  |
| **Physical activity** | Yes (Active) | Frequency of taking part in moderate or vigorous physical activity:  1.Everyday  2.More than once a week  3.Once per week | | | Number of times per week the respondent “works out” or “exercises”:  1.>=1 | Number of days of vigorous/ moderate  physical activity for at least 10 minutes every  week:  1.1-7 days |
|  | No (Inactive) | 4.One to three times a month  5.Hardly ever or never taking part in vigorous/moderate physical activity | | | 0.No | 0.No |
| **Current smoking** | No | Do not smoke at the present time | | | | |
|  | Yes | Currently smokers | | | | |
| **Drinking status** | Less than weekly drinking | Frequency of drinking:  0. 0 day/week | | Whether drinks weekly or had an alcoholic drink during the last 7 days:  0.no | The highest frequency of drinking during the last year:  0.None or less than once a month  1.One to several times a month | Frequency of drinking behaviour during the last year:  0.None or doesn’t drink  1.Once a month,  2.2 to 3 days a month |
|  | Weekly drinking or more | 1-7.1-7days/week | | 1.Yes | 2.One to several times a week  3.Most days of the week  4.Every day of the week | 3.Once a week  4.2 to 3 days a week  5.4 to 6 days a week  6.Daily  7.Twice a day  8.More than twice a day. |
| **Hypertension** | No | Respondent reported having no hypertension | | | | |
|  | Yes | Respondent reported having hypertension | | | | |
| **Psychological disorders** | No | Respondent reported having no psychological disorders(such as emotional, nervous, or psychiatric problems). | | | | |
|  | Yes | Respondent reported having emotional, nervous, or psychiatric problems. | | | | |
| **Diabetes** | No | Respondent reported having no diabetes | | | | |
|  | Yes | Respondent reported having diabetes | | | | |
| **Cardiovascular diseases** | No | Respondent reported having no stroke and heart disease (including heart attack, coronary heart disease, angina, congestive heart failure, or other heart problems) | | | | |
|  | Yes | Respondent reported having stroke or heart disease | | | | |
| **Chronic lung diseases** | No | Respondent reported having no chronic lung disease (such as chronic bronchitis or emphysema) | | | | |
|  | Yes | Respondent reported having chronic lung disease | | | | |
| **Cancer** | No | Respondent reported having no cancer (excluding minor skin cancers) | | | | |
|  | Yes | Respondent reported having cancer | | | | |

HRS, the US Health and Retirement Study; ELSA, English Longitudinal Study on Ageing; SHARE, Survey of Health, Ageing and Retirement in Europe; KLoSA, Korean Longitudinal Study of Aging; CHARLS, China Health and Retirement Longitudinal Study.

**Table S2.** Study-specific Spearman correlations between education and total household wealth.

| **Study** | **Spearman correlations of education-total household wealth** | ***P* value** |
| --- | --- | --- |
| HRS | 0.34 | <.0001 |
| ELSA | 0.36 | <.0001 |
| SHARE | 0.20 | <.0001 |
| CHARLS | 0.15 | 0.0074 |
| KLoSA | 0.26 | <.0001 |

HRS, the US Health and Retirement Study; ELSA, English Longitudinal Study on Ageing; SHARE, Survey of Health, Ageing and Retirement in Europe; CHARLS, China Health and Retirement Longitudinal Study; KLoSA, Korean Longitudinal Study of Aging.

**Table S3.** Characteristics of participants in each study by country.

| **Country** | **Study** | **Baseline year** | **Waves** | **No. of participants**  **(Total/female)** | **Age at the baseline**  **(Mean (SD))^1^** |
| --- | --- | --- | --- | --- | --- |
| Austria | SHARE | 2004-2005 | Wave1-Wave8 | 188/109 | 69.46 (8.28) |
| Belgium | SHARE | 2004-2005 | Wave1-Wave8 | 327/184 | 69.63 (8.41) |
| China | CHARLS | 2011-2012 | Wave1-Wave5 | 306/213 | 62.75 (8.44) |
| Czech Republic | SHARE | 2006-2008 | Wave2-Wave8 | 285/169 | 69.01 (7.23) |
| Denmark | SHARE | 2004-2005 | Wave1-Wave8 | 166/89 | 67.54 (7.73) |
| Estonia | SHARE | 2011-2012 | Wave4-Wave8 | 192/127 | 70.21 (8.38) |
| France | SHARE | 2004-2005 | Wave1-Wave8 | 231/124 | 68.74 (8.23) |
| Germany | SHARE | 2004-2005 | Wave1-Wave8 | 155/80 | 69.74 (6.94) |
| Italy | SHARE | 2004-2005 | Wave1-Wave8 | 243/135 | 70.77 (6.65) |
| Netherlands | SHARE | 2004-2005 | Wave1-Wave8 | 80/40 | 68.70 (6.91) |
| South Korea | KLOSA | 2006-2007 | Wave1-Wave8 | 1048/630 | 68.40 (8.66) |
|  | KLOSA _refreshment1 | 2014-2015 | Wave5-Wave8 | 10/3 | 59.20 (7.63) |
| Slovenia | SHARE | 2011-2012 | Wave4-Wave8 | 82/43 | 69.12 (7.79) |
| Spain | SHARE | 2004-2005 | Wave1-Wave8 | 286/153 | 72.39 (8.04) |
| Sweden | SHARE | 2004-2005 | Wave1-Wave8 | 211/117 | 71.17 (7.00) |
| Switzerland | SHARE | 2004-2005 | Wave1-Wave8 | 152/74 | 69.68 (8.31) |
| UK | ELSA | 2002-2003 | Wave1-Wave9 | 1039/540 | 70.27 (7.54) |
|  | ELSA_refreshment1 | 2006-2007 | Wave3-Wave9 | 83/43 | 57.54 (2.12) |
|  | ELSA_refreshment2 | 2008-2009 | Wave4-Wave9 | 165/75 | 67.59 (6.55) |
| US | HRS | 2004-2005 | Wave7-Wave15 | 2935/1711 | 69.42 (8.22) |
|  | HRS_MBB cohort | 2010-2011 | Wave10-Wave15 | 334/182 | 57.19 (3.46) |
| Total | / | / | / | **8518/4841** | **68.66 (8.42)** |

^1^ Age was assessed at the survey two years after participants first reported the diagnosis of any disease of interest. HRS, the US Health and Retirement Study; ELSA, English Longitudinal Study on Ageing; SHARE, Survey of Health, Ageing and Retirement in Europe; CHARLS, China Health and Retirement Longitudinal Study; KLoSA, Korean Longitudinal Study of Aging. ELSA kept refreshing its samples in addition to the initial cohort. HRS began to recruit Mid Baby Boomer (MBB) cohort (born 1954 to 1959) in 2010.

**Table S4.** Characteristics of participants by health behaviour status before and after diagnosis of NCDs and maintenance (N = 8,518).

| **Characteristics*** | **Physically active before and after disease diagnosis (n=5,990)** | | | **Physically inactive before but physically active after disease diagnosis (n=639)** | | | **Non-smoker before and after disease diagnosis (n=7,037)** | | | **Quit smoking after disease diagnosis (n=551)** | | |
| --- | --- | --- | --- | --- | --- | --- | --- | --- | --- | --- | --- | --- |
|  | Maintaining physical activity during follow-up (n=4,845) | Becoming physically inactive during follow-up (n=1,145) | P value | Maintaining physical activity during follow-up (n=307) | Becoming physically inactive during follow-up (n=332) | P value | Maintaining non-smoking during follow-up (n=6,972) | Initiating smoking during follow-up (n=65) | P value | Maintaining non-smoking during follow-up (n=462) | Relapsing smoking during follow-up (n=89) | P value |
| **Age (years), mean (SD)** | 67.8 (7.9) | 70.7 (8.8) | <0.001 | 67.9 (8.3) | 68.7 (8.5) | 0.161 | 69.4 (8.4) | 67.0 (8.7) | 0.037 | 66.3 (7.6) | 63.7 (7.9) | 0.004 |
| **Sex, n (%)** |  |  | 0.009 |  |  | 0.549 |  |  | <0.001 |  |  | 0.540 |
| Male | 2,367 (48.9) | 510 (44.5) |  | 118 (38.4) | 120 (36.1) |  | 2,796 (40.1) | 44 (67.7) |  | 291 (63.0) | 53 (59.6) |  |
| Female | 2,478 (51.1) | 635 (55.5) |  | 189 (61.6) | 212 (63.9) |  | 4,176 (59.9) | 21 (32.3) |  | 171 (37.0) | 36 (40.4) |  |
| **Educational level** |  |  | <0.001 |  |  | 0.006 |  |  | 0.211 |  |  | 0.524 |
| Primary | 1,328 (27.4) | 429 (37.5) |  | 123 (40.1) | 175 (52.7) |  | 2,590 (37.1) | 22 (33.8) |  | 178 (38.5) | 37 (41.6) |  |
| Secondary | 1,809 (37.3) | 466 (40.7) |  | 124 (40.4) | 109 (32.8) |  | 2,409 (34.6) | 29 (44.6) |  | 190 (41.1) | 31 (34.8) |  |
| Tertiary | 1,708 (35.3) | 250 (21.8) |  | 60 (19.5) | 48 (14.5) |  | 1,973 (28.3) | 14 (21.5) |  | 94 (20.3) | 21 (23.6) |  |
| **Total household wealth** |  |  | <0.001 |  |  | 0.322 |  |  | 0.808 |  |  | 0.254 |
| Quartile1 (lowest) | 896 (18.5) | 314 (27.4) |  | 78 (25.4) | 106 (31.9) |  | 1,446 (20.7) | 14 (21.5) |  | 118 (25.5) | 30 (33.7) |  |
| Quartile2 | 1,133 (23.4) | 311 (27.2) |  | 89 (29.0) | 89 (26.8) |  | 1,810 (26.0) | 20 (30.8) |  | 122 (26.4) | 26 (29.2) |  |
| Quartile3 | 1,283 (26.5) | 263 (23.0) |  | 76 (24.8) | 71 (21.4) |  | 1,787 (25.6) | 15 (23.1) |  | 109 (23.6) | 17 (19.1) |  |
| Quartile4 (highest) | 1,533 (31.6) | 257 (22.4) |  | 64 (20.8) | 66 (19.9) |  | 1,929 (27.7) | 16 (24.6) |  | 113 (24.5) | 16 (18.0) |  |
| **Marital status** |  |  | <0.001 |  |  | 0.385 |  |  | 0.352 |  |  | 0.439 |
| Married/partnered | 3,414 (70.5) | 736 (64.3) |  | 199 (64.8) | 215 (64.8) |  | 4,743 (68.0) | 48 (73.8) |  | 336 (72.7) | 68 (76.4) |  |
| Separated/divorced | 533 (11.0) | 114 (10.0) |  | 28 (9.1) | 26 (7.8) |  | 598 (8.6) | 7 (10.8) |  | 55 (11.9) | 13 (14.6) |  |
| Widowed | 655 (13.5) | 250 (21.8) |  | 63 (20.5) | 82 (24.7) |  | 1,355 (19.4) | 7 (10.8) |  | 49 (10.6) | 5 (5.6) |  |
| Never married | 224 (4.6) | 43 (3.8) |  | 14 (4.6) | 9 (2.7) |  | 256 (3.7) | 2 (3.1) |  | 21 (4.5) | 3 (3.4) |  |
| Unknown | 19 (0.4) | 2 (0.2) |  | 3 (1.0) | 0 (0.0) |  | 20 (0.3) | 1 (1.5) |  | 1 (0.2) | 0 (0.0) |  |
| **Body mass index** |  |  | <0.001 |  |  | 0.456 |  |  | 0.703 |  |  | 0.619 |
| Underweight | 43 (0.9) | 21 (1.8) |  | 4 (1.3) | 5 (1.5) |  | 118 (1.7) | 1 (1.5) |  | 8 (1.7) | 3 (3.4) |  |
| Normal weight | 1,244 (25.7) | 312 (27.2) |  | 92 (30.0) | 117 (35.2) |  | 1,952 (28.0) | 21 (32.3) |  | 162 (35.1) | 33 (37.1) |  |
| Overweight | 1,771 (36.6) | 345 (30.1) |  | 93 (30.3) | 90 (27.1) |  | 2,291 (32.9) | 24 (36.9) |  | 146 (31.6) | 24 (27.0) |  |
| Obesity | 1,225 (25.3) | 306 (26.7) |  | 80 (26.1) | 76 (22.9) |  | 1,774 (25.4) | 13 (20.0) |  | 88 (19.0) | 19 (21.3) |  |
| Unknown | 562 (11.6) | 161 (14.1) |  | 38 (12.4) | 44 (13.3) |  | 837 (12.0) | 6 (9.2) |  | 58 (12.6) | 10 (11.2) |  |
| **Drinking status** |  |  | <0.001 |  |  | 0.002 |  |  | 0.102 |  |  | 0.367 |
| Less than weekly drinking | 2,140 (44.2) | 694 (60.6) |  | 205 (66.8) | 254 (76.5) |  | 4,054 (58.1) | 31 (47.7) |  | 273 (59.1) | 47 (52.8) |  |
| Weekly drinking or more | 2,612 (53.9) | 418 (36.5) |  | 99 (32.2) | 70 (21.1) |  | 2,774 (39.8) | 32 (49.2) |  | 178 (38.5) | 38 (42.7) |  |
| Unknown | 93 (1.9) | 33 (2.9) |  | 3 (1.0) | 8 (2.4) |  | 144 (2.1) | 2 (3.1) |  | 11 (2.4) | 4 (4.5) |  |
| **Hypertension** | 2,950 (60.9) | 770 (67.2) | <0.001 | 209 (68.1) | 230 (69.3) | 0.744 | 4,474 (64.2) | 32 (49.2) | 0.012 | 277 (60.0) | 53 (59.6) | 0.962 |
| **Psychological disorders** | 706 (14.6) | 204 (17.8) | 0.006 | 49 (16.0) | 60 (18.1) | 0.478 | 1,037 (14.9) | 7 (10.8) | 0.353 | 67 (14.5) | 13 (14.6) | 0.980 |
| **Major NCDs** |  |  |  |  |  |  |  |  |  |  |  |  |
| Diabetes | 1,511 (31.2) | 393 (34.4) | 0.039 | 118 (38.4) | 129 (38.9) | 0.914 | 2,358 (33.9) | 21 (32.3) | 0.793 | 133 (28.8) | 35 (39.3) | 0.048 |
| Cardiovascular diseases | 2,027 (41.8) | 536 (46.9) | 0.021 | 105 (34.4) | 139 (42.0) | 0.050 | 3,107 (44.7) | 23 (35.4) | 0.133 | 221 (48.1) | 33 (37.1) | 0.055 |
| Chronic lung diseases | 639 (13.2) | 161 (14.1) | 0.429 | 44 (14.3) | 36 (10.8) | 0.183 | 797 (11.4) | 10 (15.4) | 0.319 | 92 (19.9) | 25 (28.1) | 0.084 |
| Cancer | 1,117 (23.1) | 220 (19.2) | 0.005 | 62 (20.2) | 64 (19.3) | 0.771 | 1,472 (21.1) | 16 (24.6) | 0.491 | 111 (24.0) | 11 (12.4) | 0.015 |

Data are n (%) unless otherwise indicated. Characteristics were assessed at the first two-year post-diagnosis of the NCDs. T-test and Chi-squared test were used to compare differences across groups. NCDs, non-communicable diseases.

**Table S5.** Associations between the summed SES score and maintenance of behaviours in each study.

| **Summed SES score** | **HRS (n=3,269)** | **ELSA (n=1,287)** | **SHARE (n=2,598)** | **CHARLS (n=306)** | **KLoSA (n=1,058)** |
| --- | --- | --- | --- | --- | --- |
|  | **Becoming physically inactive during follow-up** | | | | |
| Low (0) | 3.08 (2.05, 4.63) | 14.22 (5.34, 37.81) | 4.06 (2.18, 7.57) | - | 2.93 (1.05, 8.13) |
| Lower-middle (1-2) | 3.26 (2.43, 4.37) | 5.88 (2.31, 14.96) | 2.84 (1.61, 5.03) | - | 1.64 (0.8, 3.37) |
| Upper-middle (3-4) | 1.63 (1.22, 2.18) | 3.57 (1.39, 9.16) | 1.86 (1.04, 3.34) | - | 1.17 (0.58, 2.37) |
| High (5) | 1.00 (Ref) | 1.00 (Ref) | 1.00 (Ref) | 1.00 (Ref) | 1.00 (Ref) |
|  | **Becoming smokers during follow-up** | | | | |
| Low (0) | 2.89 (0.82, 10.16) | 1.84 (0.34, 10.05) | 0.86 (0.19, 3.92) | - | - |
| Lower-middle (1-2) | 2.57 (0.96, 6.9) | 1.09 (0.23, 5.22) | 1.72 (0.59, 5.03) | - | - |
| Upper-middle (3-4) | 1.62 (0.59, 4.46) | 0.37 (0.06, 2.27) | 2.02 (0.7, 5.87) | - | - |
| High (5) | 1.00 (Ref) | 1.00 (Ref) | 1.00 (Ref) | 1.00 (Ref) | 1.00 (Ref) |

Data are odds ratios and 95%CI, OR>1 indicates higher odds of relapsing unfavourable behaviours. NCDs, non-communicable diseases. Ref, reference. Socioeconomic status was constructed as the summed score (ranging from 0 to 5) of educational level (0, 1, or 2) and THW quartiles (0, 1, 2, or 3), and categorized into four groups of low (0), lower-middle (1-2), upper-middle (3-4) and high SES (5). Models were adjusted for age and sex; HRS, the US Health and Retirement Study; ELSA, English Longitudinal Study on Ageing; SHARE, Survey of Health, Ageing and Retirement in Europe; CHARLS, China Health and Retirement Longitudinal Study; KLoSA, Korean Longitudinal Study of Aging.**Table S6.** Subgroup analysis of associations between SES and maintenance of favourable behaviours by age and sex.

|  |  | **Summed SES score** | | | |
| --- | --- | --- | --- | --- | --- |
|  |  | Low | Lower-middle | Upper-middle | High |
|  |  | **Becoming physically inactive during 4-year follow-up:** | | | |
| **Age** |  |  |  |  |  |
| 45-54 | Observed prevalence | 29.2% (7/24) | 29.3% (31/106) | 11.3% (9/80) | 17.4% (4/23) |
|  | OR (95% CI) | 1.90 (0.47, 7.67) | 1.92 (0.6, 6.12) | 0.60 (0.17, 2.16) | 1.00 (Ref) |
| 55-64 | Observed prevalence | 24.9% (46/185) | 24.9% (199/798) | 13.4% (107/800) | 5.9% (17/290) |
|  | OR (95% CI) | 5.20 (2.87, 9.42) | 5.20 (3.11, 8.72) | 2.45 (1.44, 4.16) | 1.00 (Ref) |
| 65-74 | Observed prevalence | 35.3% (78/221) | 23.4% (230/983) | 17.2% (179/1038) | 10.2% (42/412) |
|  | OR (95% CI) | 4.76 (3.11, 7.26) | 2.67 (1.88, 3.80) | 1.83 (1.28, 2.62) | 1.00 (Ref) |
| 75-85 | Observed prevalence | 40.7% (77/189) | 35.7% (227/636) | 27.3% (177/649) | 24.1% (47/195) |
|  | OR (95% CI) | 2.08 (1.33, 3.24) | 1.71 (1.18, 2.47) | 1.16 (0.80, 1.69) | 1.00 (Ref) |
| **Sex** |  |  |  |  |  |
| Male | Observed prevalence | 31.0% (71/229) | 24.4% (264/1080) | 17.6% (226/1281) | 13.1% (69/525) |
|  | OR (95% CI) | 3.16 (2.16, 4.63) | 2.19 (1.64, 2.94) | 1.43 (1.07, 1.92) | 1.00 (Ref) |
| Female | Observed prevalence | 35.1% (137/390) | 29.3% (423/1443) | 19.1% (246/1286) | 10.4% (41/395) |
|  | OR (95% CI) | 4.35 (2.95, 6.40) | 3.53 (2.50, 4.97) | 2.00 (1.40, 2.84) | 1.00 (Ref) |
|  |  | **Initiating or relapsing smoking during 4-year follow-up:** | | | |
| **Age** |  |  |  |  |  |
| 45-54 | Observed prevalence | 5.7% (2/35) | 6.3% (8/128) | 2.9% (3/103) | 0.0% (0/23) |
|  | OR (95% CI) | - | - | - | 1.00 (Ref) |
| 55-64 | Observed prevalence | 5.0% (10/199) | 2.9% (24/841) | 3.3% (26/791) | 1.4% (4/278) |
|  | OR (95% CI) | 4.01 (1.24, 13.04) | 2.29 (0.78, 6.68) | 2.41 (0.83, 6.99) | 1.00 (Ref) |
| 65-74 | Observed prevalence | 1.5% (4/273) | 2.4% (28/1190) | 1.6% (18/1106) | 1.2% (5/419) |
|  | OR (95% CI) | 1.62 (0.43, 6.15) | 2.54 (0.97, 6.67) | 1.49 (0.55, 4.05) | 1.00 (Ref) |
| 75-85 | Observed prevalence | 0.6% (5/315) | 0.8% (7/910) | 1.0% (8/772) | 0.9% (2/215) |
|  | OR (95% CI) | 2.56 (0.48, 13.65) | 1.02 (0.21, 5.00) | 1.25 (0.26, 5.95) | 1.00 (Ref) |
| **Sex** |  |  |  |  |  |
| Male | Observed prevalence | 5.5% (14/256) | 3.6% (40/1102) | 2.8% (36/1309) | 1.4% (7/517) |
|  | OR (95% CI) | 4.15 (1.65, 10.44) | 2.80 (1.25, 6.31) | 2.06 (0.91, 4.65) | 1.00 (Ref) |
| Female | Observed prevalence | 1.2% (7/566) | 1.4% (27/1957) | 1.3% (19/1463) | 1.0% (4/418) |
|  | OR (95% CI) | 1.56 (0.45, 5.37) | 1.49 (0.52, 4.3) | 1.4 (0.47, 4.14) | 1.00 (Ref) |

NCDs, non-communicable diseases. Socioeconomic status was constructed as the summed score (ranging from 0 to 5) of educational level (0, 1, or 2) and THW quartiles (0, 1, 2, or 3), and categorized into four groups of low (0), lower-middle (1-2), upper-middle (3-4) and high SES (5).

**Table S7.** Subgroup analysis of associations between SES and maintenance of favourable behaviours by pre‑diagnosis behaviours.

|  | **Physically active before and after disease diagnosis (n=5,990)^1^** | **Physically inactive before but physically active after disease diagnosis (n=639)^2^** |  | **Non-smokers before and after disease diagnosis (n=7,037)^3^** | **Quit smoking after disease diagnosis (n=551)^4^** |
| --- | --- | --- | --- | --- | --- |
| **Educational level** |  |  |  |  |  |
| Primary | 1.74 (1.44, 2.11) | 1.57 (0.96, 2.56) |  | 1.26 (0.61, 2.62) | 1.14 (0.58, 2.22) |
| Secondary | 1.59 (1.33, 1.9) | 1.02 (0.63, 1.65) |  | 1.54 (0.79, 3.03) | 0.73 (0.38, 1.40) |
| Tertiary | 1.00 (Ref) | 1.00 (Ref) |  | 1.00 (Ref) | 1.00 (Ref) |
| **Total household wealth** |  |  |  |  |  |
| Quartile 1 (Lowest) | 1.82 (1.49, 2.23) | 1.21 (0.75, 1.97) |  | 1.31 (0.61, 2.83) | 1.54 (0.75, 3.17) |
| Quartile 2 | 1.43 (1.18, 1.73) | 0.87 (0.54, 1.40) |  | 1.51 (0.76, 3.01) | 1.43 (0.71, 2.89) |
| Quartile 3 | 1.08 (0.89, 1.31) | 0.85 (0.53, 1.39) |  | 1.06 (0.52, 2.17) | 1.05 (0.50, 2.23) |
| Quartile 4 (Highest) | 1.00 (Ref) | 1.00 (Ref) |  | 1.00 (Ref) | 1.00 (Ref) |
| **Summed SES-score** |  |  |  |  |  |
| Low (0) | 2.56 (2.01, 3.27) | 1.68 (0.78, 3.61) |  | 1.52 (0.48, 4.86) | 2.38 (0.77, 7.41) |
| Lower-middle (1-2) | 1.59 (1.25, 2.03) | 1.27 (0.64, 2.52) |  | 1.77 (0.72, 4.36) | 1.45 (0.52, 4.02) |
| Upper-middle (3-4) | 0.93 (0.81, 1.07) | 0.95 (0.47, 1.91) |  | 1.52 (0.62, 3.75) | 1.47 (0.52, 4.1) |
| High (5) | 1.00 (Ref) | 1.00 (Ref) |  | 1.00 (Ref) | 1.00 (Ref) |

Data are odds ratios and 95% CI. NCDs, non-communicable diseases. Ref, reference. Socioeconomic status was constructed as the summed score (ranging from 0 to 5) of educational level (0, 1, or 2) and THW quartiles (0, 1, 2, or 3), and categorized into four groups of low (0), lower-middle (1-2), upper-middle (3-4) and high SES (5).

^1^ Odds ratio of participants who ceased physical activity in those who continued physical activity after the first two-year of disease diagnosis. ^2^ Odds ratio of participants who relapsed physical inactivity in those who initiated physical activity after the first two-year of disease diagnosis. ^3^ Odds ratio of participants who initiated smoking in those who kept non-smoking after the first two-year of disease diagnosis. ^4^ Odds ratio of participants who relapsed smoking in those who ceased smoking after the first two-year of disease diagnosis. Reference groups: tertiary educational level, highest total household wealth and high socioeconomic status. Models were adjusted for age at baseline, sex, and study.

**Table S8.** Sensitivity analysis for the associations between SES and maintenance of favourable behaviours by individual NCDs.

|  | **Individual NCDs** | | | |
| --- | --- | --- | --- | --- |
|  | Diabetes | Cardiovascular disease | Chronic lung disease | Cancer |
| **Summed SES score** | **Becoming physically inactive during 4-year follow-up:**  Prevalence (N cases/N total) | | | |
| Low (0) | 30.3% (69/228) | 34.3% (99/289) | 29.3% (29/99) | 39.3% (35/89) |
| Lower-middle (1-2) | 28.6% (262/915) | 29.8% (322/1080) | 24.0% (91/380) | 26.2% (116/443) |
| Upper-middle (3-4) | 21.6% (167/772) | 19.1% (205/1075) | 18.4% (57/310) | 17.1% (110/644) |
| High (5) | 10.2% (24/236) | 13.5% (49/363) | 22.0% (20/91) | 8.0% (23/287) |
|  | Odds ratio (95% confidence interval) | | | |
| Low (0) | 3.22 (1.92, 5.38) | 3.21 (2.17, 4.75) | 1.52 (0.78, 2.95) | 7.24 (3.94, 13.31) |
| Lower-middle (1-2) | 3.23 (2.06, 5.06) | 2.76 (1.98, 3.84) | 1.17 (0.67, 2.03) | 3.96 (2.45, 6.38) |
| Upper-middle (3-4) | 2.27 (1.44, 3.59) | 1.49 (1.06, 2.10) | 0.83 (0.47, 1.49) | 2.31 (1.44, 3.71) |
| High (5) | 1.00 (Ref) | 1.00 (Ref) | 1.00 (Ref) | 1.00 (Ref) |
|  | **Initiating or relapsing smoking during 4-year follow-up:**  Prevalence (N cases/N total) | | | |
| Low (0) | 1.9% (6/314) | 2.6% (11/416) | 6.5% (8/124) | 1.7% (2/117) |
| Lower-middle (1-2) | 2.5% (28/1137) | 1.8% (25/1366) | 3.0% (12/406) | 1.5% (8/527) |
| Upper-middle (3-4) | 2.2% (19/853) | 1.4% (17/1217) | 4.2% (13/309) | 1.9% (13/682) |
| High (5) | 1.2% (3/243) | 0.8% (3/385) | 2.4% (2/85) | 1.4% (4/284) |
|  | Odds ratio (95% confidence interval) | | | |
| Low (0) | 2.52 (0.62, 10.33) | 4.34 (1.19, 15.82) | 3.04 (0.61, 15.09) | 1.44 (0.26, 8.04) |
| Lower-middle (1-2) | 3.00 (0.89, 10.04) | 2.80 (0.83, 9.37) | 1.31 (0.28, 6.05) | 1.18 (0.35, 3.99) |
| Upper-middle (3-4) | 2.20 (0.64, 7.55) | 1.94 (0.56, 6.69) | 1.71 (0.37, 7.84) | 1.39 (0.45, 4.31) |
| High (5) | 1.00 (Ref) | 1.00 (Ref) | 1.00 (Ref) | 1.00 (Ref) |
|  | **Relapsing smoking during 4-year follow-up:**  Prevalence (N cases/ N total) | | | |
| Low (0) | 27.3% (6/22) | 18.6% (8/43) | 60.0% (6/10) | 7.7% (1/13) |
| Lower-middle (1-2) | 21.0% (17/81) | 12.3% (14/114) | 13.13% (8/60) | 5.9% (3/51) |
| Upper-middle (3-4) | 21.2% (11/52) | 10.8% (6/74) | 22.7% (10/44) | 12.2% (6/49) |
| High (5) | 7.7% (1/13) | 13.0% (3/23) | 33.3% (1/3) | 11.1% (1/9) |
|  | Odds ratio (95% confidence interval) | | | |
| Low (0) | 5.42 (0.55, 53.45) | 1.29 (0.30, 5.55) | 3.89 (0.23, 65.99) | 0.76 (0.04, 14.34) |
| Lower-middle (1-2) | 3.66 (0.43, 31.05) | 0.78 (0.20, 3.06) | 0.46 (0.03, 6.16) | 0.52 (0.05, 5.81) |
| Upper-middle (3-4) | 3.46 (0.40, 30.10) | 0.75 (0.18, 3.14) | 0.76 (0.06, 10.02) | 1.08 (0.11, 10.38) |
| High (5) | 1.00 (Ref) | 1.00 (Ref) | 1.00 (Ref) | 1.00 (Ref) |

NCDs, non-communicable diseases. Ref, reference. Socioeconomic status was constructed as the summed score (ranging from 0 to 5) of educational level (0, 1, or 2) and total household wealth quartiles (0, 1, 2, or 3), and categorized into four groups of low (0), lower-middle (1-2), upper-middle (3-4) and high SES (5).

**Table S9.** Sensitivity analysis for the shorter- and longer-term associations between SES and maintenance of favourable behaviours.

| **Socioeconomic indicators** | **Shorter-term^1^** | | **Longer-term^2^** | |
| --- | --- | --- | --- | --- |
|  | Participants with physical activity data two years after baseline^3^ (n=5,838) | Participants with tobacco use data two years after baseline^4^ (n=6,758) | Participants with physical activity data four years after baseline^5^ (n=5,763) | Participants with tobacco use data four years after baseline^6^ (n=6,288) |
| **Educational level** | |  |  |  |
| Primary | 1.93 (1.55, 2.40) | 0.98 (0.51, 1.86) | 1.85 (1.50, 2.28) | 0.88 (0.47, 1.64) |
| Secondary | 1.50 (1.21, 1.86) | 0.93 (0.49, 1.75) | 1.51 (1.24, 1.85) | 1.16 (0.66, 2.03) |
| Tertiary | 1.00 (Ref) | 1.00 (Ref) | 1.00 (Ref) | 1.00 (Ref) |
| **Total household wealth** | |  |  |  |
| Quartile 1 (lowest) | 2.85 (0.95, 8.51) | 2.85 (0.95, 8.51) | 2.85 (0.95, 8.51) | 2.85 (0.95, 8.51) |
| Quartile 2 | 2.38 (0.96, 5.86) | 2.38 (0.96, 5.86) | 2.38 (0.96, 5.86) | 2.38 (0.96, 5.86) |
| Quartile 3 | 1.89 (0.77, 4.61) | 1.89 (0.77, 4.61) | 1.89 (0.77, 4.61) | 1.89 (0.77, 4.61) |
| Quartile 4 (highest) | 1.00 (Ref) | 1.00 (Ref) | 1.00 (Ref) | 1.00 (Ref) |
| **Summed SES score** | | |  |  |
| Low (0) | 3.20 (2.22, 4.61) | 4.73 (1.59, 14.06) | 2.26 (1.58, 3.23) | 2.85 (0.95, 8.51) |
| Lower-middle (1-2) | 2.34 (1.73, 3.17) | 2.28 (0.85, 6.11) | 2.31 (1.74, 3.07) | 2.38 (0.96, 5.86) |
| Upper-middle (3-4) | 1.52 (1.12, 2.07) | 2.00 (0.76, 5.29) | 1.40 (1.05, 1.87) | 1.89 (0.77, 4.61) |
| High (5) | 1.00 (Ref) | 1.00 (Ref) | 1.00 (Ref) | 1.00 (Ref) |

Data are odds ratios and 95%CI. NCDs, non-communicable diseases. Ref, reference. Socioeconomic status was constructed as the summed score (ranging from 0 to 5) of educational level (0, 1, or 2) and THW quartiles (0, 1, 2, or 3), and categorized into four groups of low (0), lower-middle (1-2), upper-middle (3-4) and high SES (5).

^1^ Short-term means the observation of the fourth year (two years after the baseline) in this study.

^2^ Long-term means the observation of the sixth year (four years after the baseline) in this study.

^3^ OR>1 indicates higher odds of becoming physically inactive in the second year of baseline.

^4^ OR>1 indicates higher odds of becoming smokers in the second year of baseline.

^5^ OR>1 indicates higher odds of becoming physically inactive in the fourth year of baseline.

^6^ OR>1 indicates higher odds of becoming smokers in the fourth year of baseline.

**Fig. S1.** Heatmaps of the associations of the 12 combinations of SES with maintenance of favourable behaviours. Data are OR (95% CI). A darker color in a gradient from white to red shows higher odds of corresponding outcomes. A darker color in a gradient from white to green shows lower odds of corresponding outcomes. Six maps shows the results in six different population:1A) participants continued physical activity after the first two-year of disease diagnosis; 2A) participants initiated physical activity after the first two-year of disease diagnosis; 3A) all participants with physically active after the first two-year of disease diagnosis; 1B) participants kept non-smoking after the first two-year of disease diagnosis, 2B) participants ceased smoking after the first two-year of disease diagnosis, and 3B) all participants with non-smoking after the first two-year of disease diagnosis. The results were adjusted for age, sex, and study. The color is in a gradient from green (OR <1) to white (OR=1, midpoint) and then to red (OR <1). NCD, non-communicable disease. ORs, odds ratios.


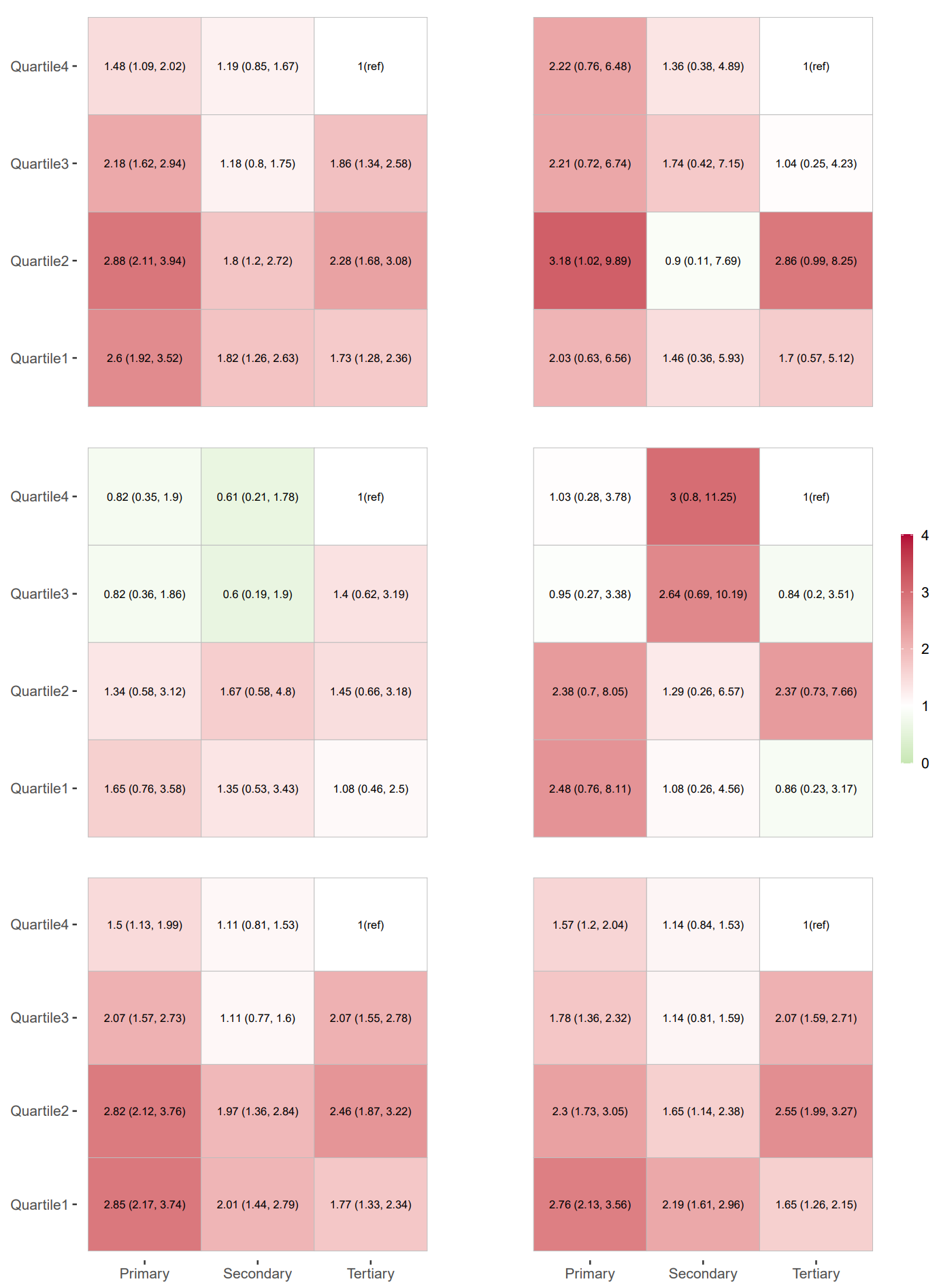


**3A) ORs for becoming physically inactive**

**2A) ORs for relapsing physically inactive**

**1A) ORs for initiating physically inactive**

**1B) ORs for initiating smoking**


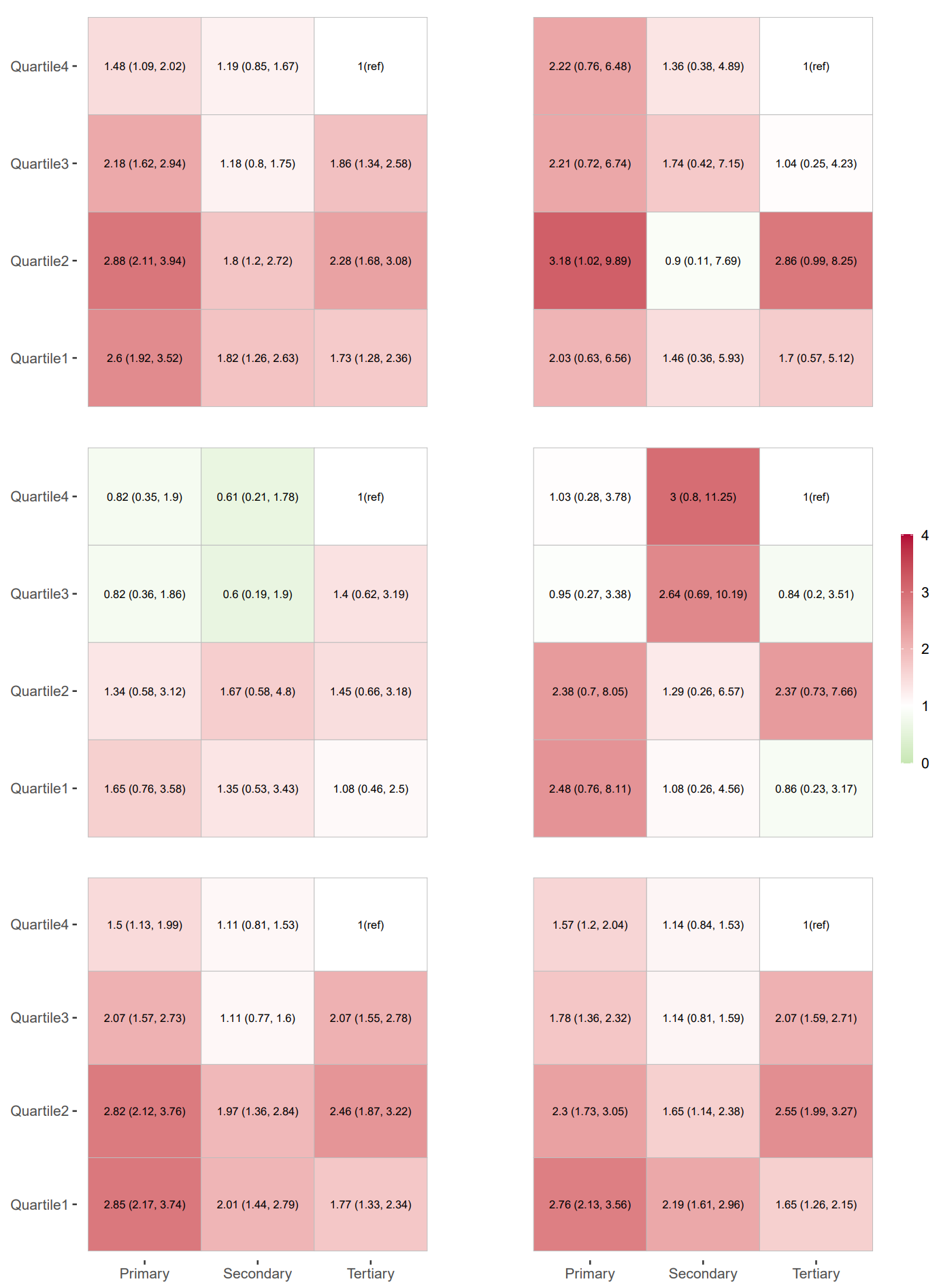


**3B) ORs for becoming smokers**

**2B) ORs for relapsing smoking**


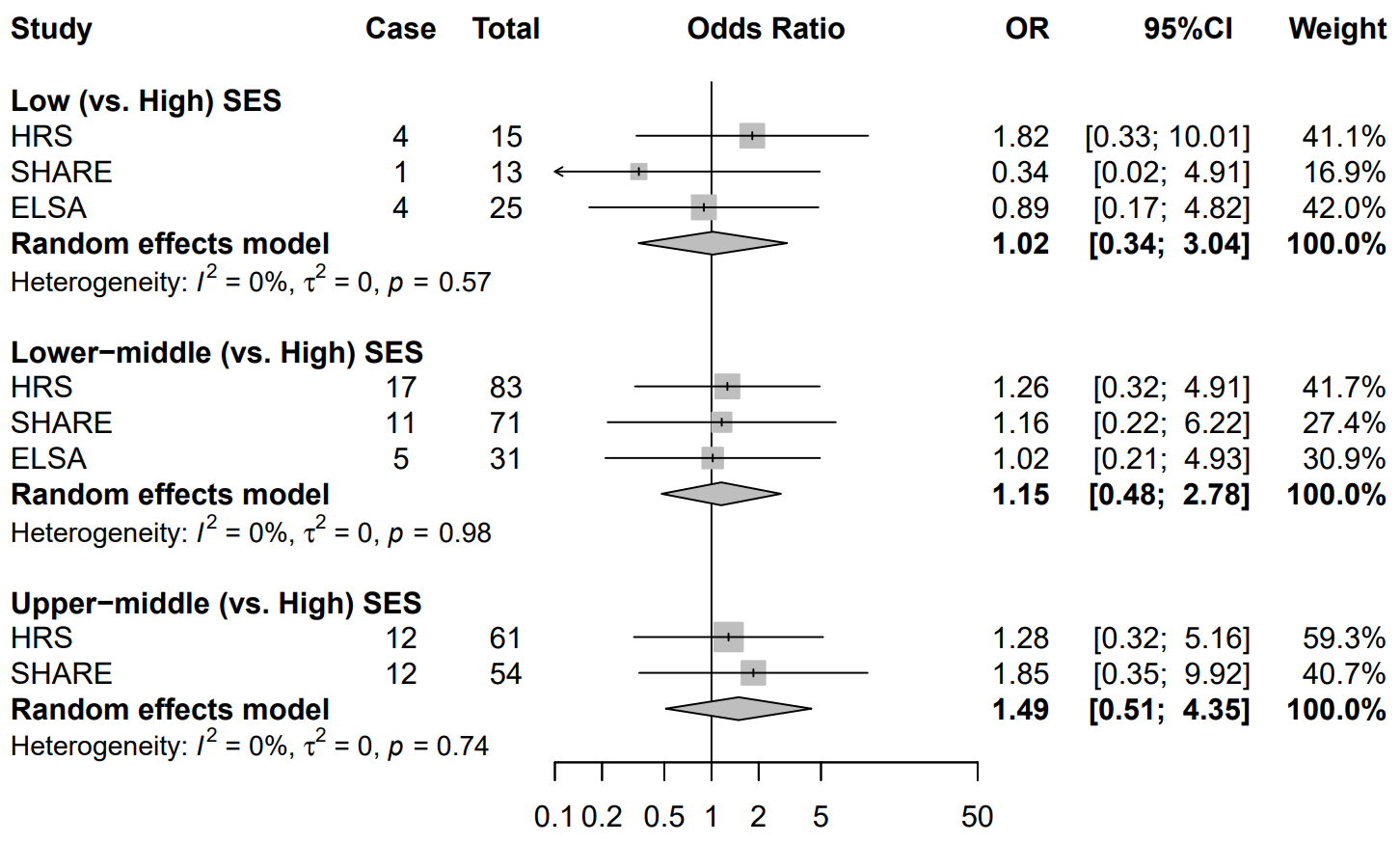
**Fig. S2**. Forest plot of study-specific ORs of the associations between SES and relapsing smoking in ex-smokers. All estimates were adjusted for age and sex. SHARE included 13 countries consisting of Austria, Belgium, Czech Republic, Denmark, Estonia, France, Germany, Italy, Netherlands, Slovenia, Spain, Sweden, and Switzerland. HRS, the US Health and Retirement Study; SHARE, Survey of Health, Ageing and Retirement in Europe; ELSA, English Longitudinal Study of Ageing; NCDs, non-communicable diseases; SES, socioeconomic status.
